# Conceptualizing Autism as a Behavioral Network: Transdiagnostic Associations with Co-occurring Psychiatric Conditions

**DOI:** 10.1007/s10802-026-01421-6

**Published:** 2026-03-14

**Authors:** Gloria T. Han, Shashwat Kala, Adam Naples, Geraldine Dawson, Susan Faja, Frederick Shic, Sara Jane Webb, Catherine A. Sugar, Shafali Jeste, Natalia Kleinhans, Katarzyna Chawarska, Raphael Bernier, James Dziura, James C. McPartland, Autism Biomarkers Consortium for Clinical Trials

**Affiliations:** 1 Vanderbilt University Medical Center, Nashville, TN USA; 2 Yale Child Study Center, Yale University School of Medicine, New Haven, CT USA; 3 Department of Psychiatry and Behavioral Sciences, Duke University, Durham, NC USA; 4 Boston Children’s Hospital, Harvard Medical School, Boston, MA USA; 5 Department of Pediatrics, Harvard Medical School, Boston, MA USA; 6 Center on Child Health, Behavior, and Development, Seattle Children’s Research Institute, Seattle, WA USA; 7 Department of Pediatrics, University of Washington, Seattle, WA USA; 8 Department of Psychiatry & Behavioral Sciences, University of Washington, Seattle, WA USA; 9 Department of Biostatistics, University of California Los Angeles, Los Angeles, CA USA; 10 Department of Psychiatry and Behavioral Sciences, University of California Los Angeles, Los Angeles, CA USA; 11 Department of Pediatrics, University of California Los Angeles, Los Angeles, CA USA; 12 Department of Radiology, University of Washington, Seattle, WA USA; 13 Department of Psychology, University of Washington, Seattle, WA USA

**Keywords:** Autism, Psychiatric comorbidity, Transdiagnostic mechanisms, Diagnostic overshadowing, Symptom overlap, Network analysis

## Abstract

Autism is characterized by marked heterogeneity in behavioral presentation and high rates of co-occurring psychiatric symptoms, which hinder diagnostic precision, personalized intervention, and long-term quality of life. Approach and withdrawal behaviors—subserved by core motivational systems underlying action and emotion—may serve as transdiagnostic processes linking autism with common co-occurring conditions in childhood. Guided by this framework, we examined how autism-related approach–withdrawal behaviors interrelate and connect to internalizing and externalizing symptoms. Using data from 280 autistic children aged 6 to 11 years enrolled in the Autism Biomarkers Consortium for Clinical Trials, we constructed a Gaussian graphical model of approach–withdrawal behaviors. Core behaviors were identified using expected influence centrality. Autism, when conceptualized as a system of interconnected approach–withdrawal behaviors, was positively associated with common co-occurring psychiatric conditions, with strongest associations observed for anxiety and attention-deficit/hyperactivity disorder. Affect regulation–related nodes were most relevant to internalizing symptoms, whereas arousal regulation and sensory nodes were uniquely related to externalizing symptoms. These findings integrate transdiagnostic theories of approach-withdrawal motivation with network analysis and highlight clinically relevant targets for diagnostic refinement and intervention.

## Introduction

Autism spectrum disorder (hereafter referred to as “autism”) is a neurodevelopmental condition characterized by differences in social communication and interaction and presence of restricted interests and repetitive behaviors (RRBs), often accompanied by differences in sensory processing (American Psychiatric Association & Association, 2013). A hallmark of autism is its heterogeneity, with wide variability in social, emotional, and functional challenges that shape the expression of co-occurring psychiatric conditions. High rates of co-occurring psychiatric conditions are well documented, with approximately 70% of autistic children experiencing at least one co-occurring psychiatric condition (Gjevik et al., 2011; Leyfer et al., 2006; Simonoff et al., 2008). Anxiety disorders (e.g., social anxiety, separation anxiety) and attention deficit hyperactivity disorder (ADHD) are the most common, followed by depression, especially in adolescence and adulthood. Longitudinally, co-occurring conditions lead to worse functional outcomes (McCauley et al., 2020) and elevated rates of suicidality, disability, and morbidity (Kõlves et al., 2021).

Understanding factors underlying the emergence of psychiatric conditions in autism is central to enhancing well-being of autistic individuals. However, identification and treatment of psychiatric conditions in autism is complicated by diagnostic overlap between core autism features (e.g., avoidance of social situations, social difficulties, attentional differences) and co-occurring symptoms (e.g., anxiety, ADHD) (Georgiades et al., 2011; Kerns & Kendall, 2014; Taurines et al., 2012). It is often challenging to differentiate inherent phenotypic variability of autism from true presence of co-occurring conditions, reducing precision in allocating appropriate psychosocial interventions to meet the psychological, social, and emotional needs of autistic individuals. Moreover, diagnostic overshadowing—the tendency for co-occurring conditions to be overlooked or misattributed to autism—further complicates recognition and treatment (Gupta et al., 2025; Rosen et al., 2018). These challenges can result in both over-diagnosis (e.g., inattention or hyperactivity attributed to ADHD rather than autism-related attentional differences) and under-diagnosis (e.g., anxiety or depression symptoms attributed solely to autism and therefore untreated), leading to potentially ineffective or inappropriate intervention (Barlattani et al., 2023; Matson & Goldin, 2014; Mazefsky et al., 2012). Despite their contributions to diagnostic imprecision, diagnostic overlap and overshadowing also suggest that autism and co-occurring conditions share interrelated component processes that may be obscured by current diagnostic practices, which rely on symptom checklists to define distinct categories. Viewing autism and co-occurring conditions at the level of shared transdiagnostic features and modeling them as a system of interrelated processes may provide greater clarity on how these conditions are connected.

## A Transdiagnostic Approach-Withdrawal Framework To Explain Autism and Co-occurring Conditions

A promising transdiagnostic framework for clarifying these relationships is grounded in approach-withdrawal motivation systems subserved by neural behavioral activation and inhibition systems (Davidson, 1998; Depue & Iacono, 1989; Gray et al., 1994). This framework has long been applied to explain psychiatric conditions: anxiety disorders are characterized by an overactive withdrawal system, leading to heightened behavioral inhibition and avoidance of feared stimuli, while depression reflects both reduced approach motivation and overactive withdrawal, producing behavioral deactivation and negative affect (Shankman & Klein, 2003). In externalizing domains, ADHD has been framed as involving underactive withdrawal or impaired response inhibition, producing impulsive, dysregulated behaviors (Barkley, 1997; Wodka et al., 2007). Conduct disorder, by contrast, has been conceptualized as an overactive approach system dominating withdrawal, leading to reward-driven disruptive behaviors, such as aggression and rule-breaking (Matthys et al., 1998). Taken together, these models demonstrate that psychiatric conditions can be parsimoniously captured through patterns of imbalance in approach versus withdrawal behaviors.

Extending this framework to autism, the social motivation hypothesis suggests reduced social approach (e.g., reduced attention to faces) and increased non-social approach (e.g., preoccupation with restricted or special interests) underlie social communication differences characterizing autism (Dawson et al., 2005). Restricted and repetitive behaviors may reflect overactive approach toward predictability and routines, while sensory hyper- and hypo-responsiveness reflect differential approach or withdrawal from specific sensory inputs (Cascio et al., 2016; Williams et al., 2021a, b). The modifier model of autism (Mundy et al., 2007) provides an especially relevant conceptual bridge: rather than viewing autism traits in isolation, it emphasizes that modifier processes—such as executive functioning, attentional control, and motivational biases—alter the expression of core autism features and contribute to co-occurring symptomatology. Within this view, approach–withdrawal tendencies are not merely comorbid dimensions; they shape how autism manifests and how co-occurring psychiatric conditions (e.g., anxiety, ADHD, depression) emerge. For example, heightened withdrawal tendencies may convert social reciprocity difficulties into clinically significant social anxiety (Bagg et al., 2024), while underactive withdrawal or overactive approach may potentiate ADHD-like impulsivity. This framing highlights the value of conceptualizing autism as embedded within broader motivational systems that also govern other psychiatric disorders.

### Characterizing Autism as a Network of Approach-Withdrawal Behaviors

From this transdiagnostic systems perspective, network analysis offers an ideal analytic approach to model these connections. Traditional categorical or latent-variable models assume that disorders arise from unobservable common causes and thus obscure the complex interdependence among symptoms (Borsboom & Cramer, 2013). In contrast, network analysis directly models the structure of observable behaviors, allowing approach–withdrawal features to be examined as interacting nodes within a dynamic system. This is especially critical in autism, where approach–withdrawal tendencies may simultaneously function as intrinsic traits, modifiers of symptom expression, and bridges to co-occurring conditions. By identifying which approach–withdrawal nodes are most central and which uniquely link autism to internalizing or externalizing symptoms, network methods can clarify diagnostic overlap, mitigate the problem of diagnostic overshadowing, and highlight precise intervention targets. In this way, network analysis is not simply a novel method but the one most conceptually aligned with the framework of approach–withdrawal as a system shaping autism and psychopathology. Recent work has applied network analysis to understand autism and co-occurring conditions such as anxiety, obsessive compulsive disorder, depression, and ADHD (Farhat et al., 2021; Montazeri et al., 2019, 2020; Ruzzano et al., 2015; van Heijst et al., 2020; Williams et al., 2021a, b). However, these studies have typically focused on one or two co-occurring conditions at a time, leaving open questions about how autism relates simultaneously to the broader range of psychiatric symptoms common in childhood.

### The Current Study

The present study addresses this gap by designating transdiagnostic approach–withdrawal behaviors as nodes of a network to assess relations between autism and co-occurring conditions across internalizing and externalizing domains in a large, well-characterized cohort of autistic children. To our knowledge, this is the first study to explicitly integrate approach–withdrawal theories of psychopathology with the modifier model of autism and apply network analysis to identify how autism-relevant approach–withdrawal behaviors link to co-occurring psychiatric conditions. In doing so, we provide a novel framework for clarifying diagnostic overlap and targeting transdiagnostic processes in intervention.

## Methods

### Participants

Participants included 280 children aged 6 to 11 years old enrolled in the Autism Biomarkers Consortium for Clinical Trials (ABC-CT), a multisite longitudinal study aimed to identify and develop objective biomarkers of social communication and functioning in autistic children. Recruitment occurred across five research centers: Yale University, Duke University, University of California Los Angeles, Boston Children’s Hospital/Harvard, and University of Washington. Autism diagnoses were confirmed using “gold-standard” procedures: the Autism Diagnostic Observation Schedule–Second Edition (ADOS-2; Lord et al. (2012), the Autism Diagnostic Interview–Revised (ADI-R; Rutter et al. (2003), and DSM-5 clinical judgment. Exclusion criteria included the presence of a known genetic condition, metabolic or mitochondrial disorder, or significant visual, auditory, or motor impairment that would limit study participation. Children were required to be stable on medications for at least 8 weeks prior to the study visit. Participants and caregivers were expected to be English-speaking. The study protocol and applicable consent documents were approved by the Yale University Institutional Review Board (IRB) which served as the Central IRB for the Autism Biomarkers Consortium for Clinical Trials (ABC-CT) (HIC#1509016477). Written informed consent was obtained from caregivers prior to study participation, and age-appropriate assent was obtained from children at the study visit prior to any study procedures were initiated. The current study utilizes data from the baseline timepoint.

Children were required to have a full-scale IQ, indexed by the General Conceptual Ability (GCA) score of the Differential Ability Scales–Second Edition (DAS-II; Elliott et al. (2007), between 60 and 150. The lower cutoff of 60 was selected to optimize inclusion of participants with intellectual disability (ID). Twenty-two children had FSIQ scores of 70 or below, 51 scored between 71 and 84, 167 between 85 and 115, and 40 scored 116 or above. This distribution reflects inclusion of children with and without ID and permits evaluation across a broad range of cognitive ability. Additional demographic characteristics are presented in Table 1.

**Table 1:** Characteristics of study participants

	Female (*N* = 65)	Male (*N* = 215)	Overall (*N* = 280)
Age (years)			
Mean (SD)	8.31 (1.59)	8.61 (1.65)	8.54 (1.64)
Median [Min, Max]	8.04 [6.04, 11.5]	8.52 [6.01, 11.6]	8.33 [6.01, 11.6]
Race			
1 = American Indian/Alaskan Native	0 (0%)	2 (0.9%)	2 (0.7%)
2 = Asian	1 (1.5%)	14 (6.5%)	15 (5.4%)
4 = Black or African American	3 (4.6%)	19 (8.8%)	22 (7.9%)
5 = White	48 (73.8%)	142 (66.0%)	190 (67.9%)
6 = More than one race	13 (20.0%)	32 (14.9%)	45 (16.1%)
8 = Other	0 (0%)	6 (2.8%)	6 (2.1%)
ADOS-2 Comparison Score			
Mean (SD)	6.94 (1.76)	7.86 (1.72)	7.65 (1.77)
Median [Min, Max]	7.00 [4.00, 10.0]	8.00 [4.00, 10.0]	8.00 [4.00, 10.0]
CASI-5 ADHD Inattentive T Score			
Mean (SD)	79.3 (14.7)	70.6 (11.1)	72.7 (12.6)
% Exceeding cutoff	74%	56%	60%
Missing	0 (0%)	4 (1.9%)	4 (1.4%)
CASI-5 ADHD Hyperactive-Impulsive T Score			
Mean (SD)	74.8 (15.8)	67.6 (13.2)	69.3 (14.1)
% Exceeding cutoff	55%	41%	45%
Missing	0 (0%)	4 (1.9%)	4 (1.4%)
CASI-5 ADHD Combined T Score			
Mean (SD)	79.9 (14.8)	71.8 (11.9)	73.7 (13.1)
% Exceeding cutoff	72%	53%	58%
Missing	0 (0%)	4 (1.9%)	4 (1.4%)
CASI-5 Generalized Anxiety T Score			
Mean (SD)	74.5 (18.6)	66.6 (14.8)	68.5 (16.1)
% Exceeding cutoff	57%	35%	41%
Missing	0 (0%)	4 (1.9%)	4 (1.4%)
CASI-5 Social Anxiety T Score			
Mean (SD)	66.3 (22.3)	59.5 (15.1)	61.1 (17.3)
% Exceeding cutoff	38%	27%	29%
Missing	0 (0%)	5 (2.3%)	5 (1.8%)
CASI-5 Separation Anxiety T Score			
Mean (SD)	60.2 (18.1)	54.9 (13.5)	56.2 (14.9)
% Exceeding cutoff	25%	11%	14%
Missing	0 (0%)	4 (1.9%)	4 (1.4%)
CASI-5 Dysthymia T Score			
Mean (SD)	71.6 (23.6)	65.7 (19.0)	67.1 (20.3)
% Exceeding cutoff	43%	33%	36%
Missing	0 (0%)	4 (1.9%)	4 (1.4%)
CASI-5 Major Depressive Episode T Score			
Mean (SD)	60.3 (17.8)	58.7 (16.5)	59.1 (16.8)
% Exceeding cutoff	20%	17%	18%
Missing	0 (0%)	4 (1.9%)	4 (1.4%)
CASI-5 Oppositional Defiant Disorder T Score			
Mean (SD)	62.8 (15.5)	59.8 (14.8)	60.5 (15.0)
% Exceeding cutoff	23%	25%	25%
Missing	0 (0%)	4 (1.9%)	4 (1.4%)
CASI-5 Conduct Disorder T Score			
Mean (SD)	55.8 (11.6)	52.9 (9.63)	53.6 (10.2)
% Exceeding cutoff	14%	7%	8%
Missing	0 (0%)	5 (2.3%)	5 (1.8%)

## Measures

### Pervasive Developmental Disorder Behavioral Inventory

The PDDBI (Cohen et al., 2003) is a parent/caregiver instrument that assesses adaptive and maladaptive behaviors in autistic children between 18 months and 12 years and 6 months of age. The PDDBI is divided into two broad behavioral dimensions: (1) Approach Withdrawal Problems and (2) Receptive/Expressive Social Communication Abilities. Given our focus on approach-withdrawal behaviors, we utilized the Approach Withdrawal Problems dimension, which is composed of seven domains: sensory/perceptual approach behaviors, ritualism/resistance to change, social pragmatic problems, semantic/pragmatic problems, arousal regulation problems, specific fears, and aggressiveness. These seven domains are subdivided into 27 clusters or facets of approach-withdrawal behavior. Each of these facets consists of four item-level questions rated on a 0 to 3 Likert scale. Average ratings of these 27 facets were used as the nodes in the network analysis. Prior work in the ABC-CT sample has demonstrated good to excellent six-week test–retest reliability (intraclass correlations ranging from 0.74 to 0.85 across relevant domains; Faja et al. (2023). This indicates that PDDBI domains, including those used in the present study, capture stable individual differences in approach–withdrawal tendencies.

### Child and Adolescent Symptom Inventory-5

The CASI-5 (Gadow & Sprafkin, 2013) is a parent/caregiver behavior rating scale used to assess symptoms of common clinical conditions, with demonstrated validity in autistic samples. Parents/caregivers rated symptom frequency on a 0 to 3 Likert scale. The CASI-5 T scores are provided for the following conditions: Asperger’s, autistic disorder, ADHD hyperactive impulsivity, ADHD inattentive, ADHD combined, separation anxiety, social anxiety, dysthymic disorder, generalized anxiety, major depressive episode, oppositional defiant disorder (ODD), and conduct disorder (CD). A T score > 70 indicates that the child exceeded the clinical cutoff for that specific psychiatric disorder. Internal consistency at baseline in the current sample was good across subscales (all α > 0.80).

### Data Analytic Plan

All analyses were conducted in R (Version 4.1.2). Descriptive analyses were first conducted to characterize the demographics and co-occurring symptomatology of the study sample.

### Network Estimation

To accommodate non-normality of the data, a non-paranormal transformation was first applied to prepare the data for network estimation (Liu et al., 2009). The PDDBI network was constructed using the *qgraph* package (Epskamp et al., 2012) in R to determine conditional independence relationships between nodes. All 27 facets of autism-related approach-withdrawal behaviors from the PDDBI were included as possible nodes in the network analysis. Because nodes that are strongly related may conflate the network structure, we used the *goldbricker* algorithm within the *networktools* R package to detect redundant nodes. This procedure resulted in 20 final nodes. As overall results were comparable, and the PDDBI is an established and standardized measure of behaviors related to autism and developmental disabilities, subsequent analyses preserved the 27 PDDBI constructs.

Regularized partial correlation networks between approach-withdrawal behaviors were estimated using the *cor_auto* function. To reduce the likelihood of spurious edges in the network, the network structure was based on L1-regularized partial correlations (Friedman et al., 2008; Tibshirani, 1996) implemented in *qgraph* using a graphical Least Absolute Shrinkage and Selection Operator (LASSO) (*glasso*) algorithm. This algorithm imposes a statistical penalty based on a λ parameter selected with the Extended Bayesian Information Criterion (EBIC) (Barber & Drton, 2015; Foygel & Drton, 2010). The EBIC hyperparameter was manually set to λ = 0.5 to fit a parsimonious network that balances many connections (γ = 0) and a network with minimal connections (γ = 1). The resulting network was plotted according to the Fruchterman and Reingold (1991) algorithm, which places the most strongly connected nodes towards the center of the network and weakly connected nodes in the periphery.

Before proceeding with additional analyses, we used the *mgm* package (Haslbeck & Waldorp, 2020) to assess whether the overall network structure differed by or was moderated by sex (Haslbeck, 2022). No evidence for moderation by sex was found. Thus, subsequent analyses were not separated based on sex. Overall stability of the network was estimated using the *bootnet* package (Epskamp et al., 2018; Epskamp & Fried, 2015).

### Network Visualization and Community Detection

In the network plot, each node represents a facet of approach-withdrawal behavior as assessed by the PDDBI. The community structure (i.e., presence of symptom sub-clusters in the network) was assessed using the *walktrap* random walk algorithm (Pons & Latapy, 2005) within the *igraph* package (Csardi & Nepusz, 2006). Simulation studies indicate that such exploratory graph analysis algorithms are useful for identifying the number of dimensions in a particular psychological construct and yield results that are comparable to more traditional methods, such as principal components and factor analysis (Golino & Epskamp, 2017). Thus, applying the community detection algorithm also provided a way to cross reference the approach-withdrawal domains of the PDDBI.

### Association between Node Centrality and Co-occurring Conditions

To assess the relationship between autism—when conceptualized as a system of symptoms (i.e., network)—and common co-occurring conditions, a two-step process was used. In Step 1, we computed zero-order correlations between each facet of approach and withdrawal yielded by the PDDBI with CASI-5 T scores of the co-occurring conditions. Zero-order correlations were used to represent how each facet of approach and withdrawal is independently related to co-occurring conditions. For example, the social withdrawal facet of the PDDBI may be particularly associated with social anxiety symptoms assessed via the CASI-5. Higher CASI-5 T scores indicate greater severity of co-occurring symptomatology. Thus, a high correlation indicates that the approach-withdrawal node is highly associated with the co-occurring condition.

In Step 2, we computed bivariate Spearman correlations to determine the relationship between node centrality (expected influence) and co-occurring symptoms. Specifically, standardized expected influence of each PDDBI node was associated with the zero-order correlations computed in Step 1 (i.e., correlations between the network nodes and each co-occurring condition computed). The strength of the association indicates to what extent centrality of approach-withdrawal behaviors (expected influence) is related to the co-occurring condition. To represent how individual nodes may be differentially or particularly related to each co-occurring condition, we also created node importance plots. In these plots, longer horizontal lines denote a stronger correlation between the designated node and co-occurring condition. “Clinically relevant” nodes were operationalized as approach-withdrawal behaviors (i.e., nodes) that were particularly central to the 27-node network *and* were highly correlated with the CASI-5 condition.

## Results

### Sample Characteristics

Participant characteristics are presented in Table 1. Participants ranged in age from 6 to 11.6 years (M = 8.54 years, SD = 1.64). Most of the sample was male (77%) and non-Hispanic (81.4%). On the Autism Diagnostic Observation Schedule, Second Edition (ADOS-2), participants had a mean calibrated severity score of 7.65 (SD = 1.77). Table 1 also presents the percentage of participants who exceeded the clinical cutoff for common co-occurring conditions.

### Autism Approach-Withdrawal Network

**Fig. 1:**
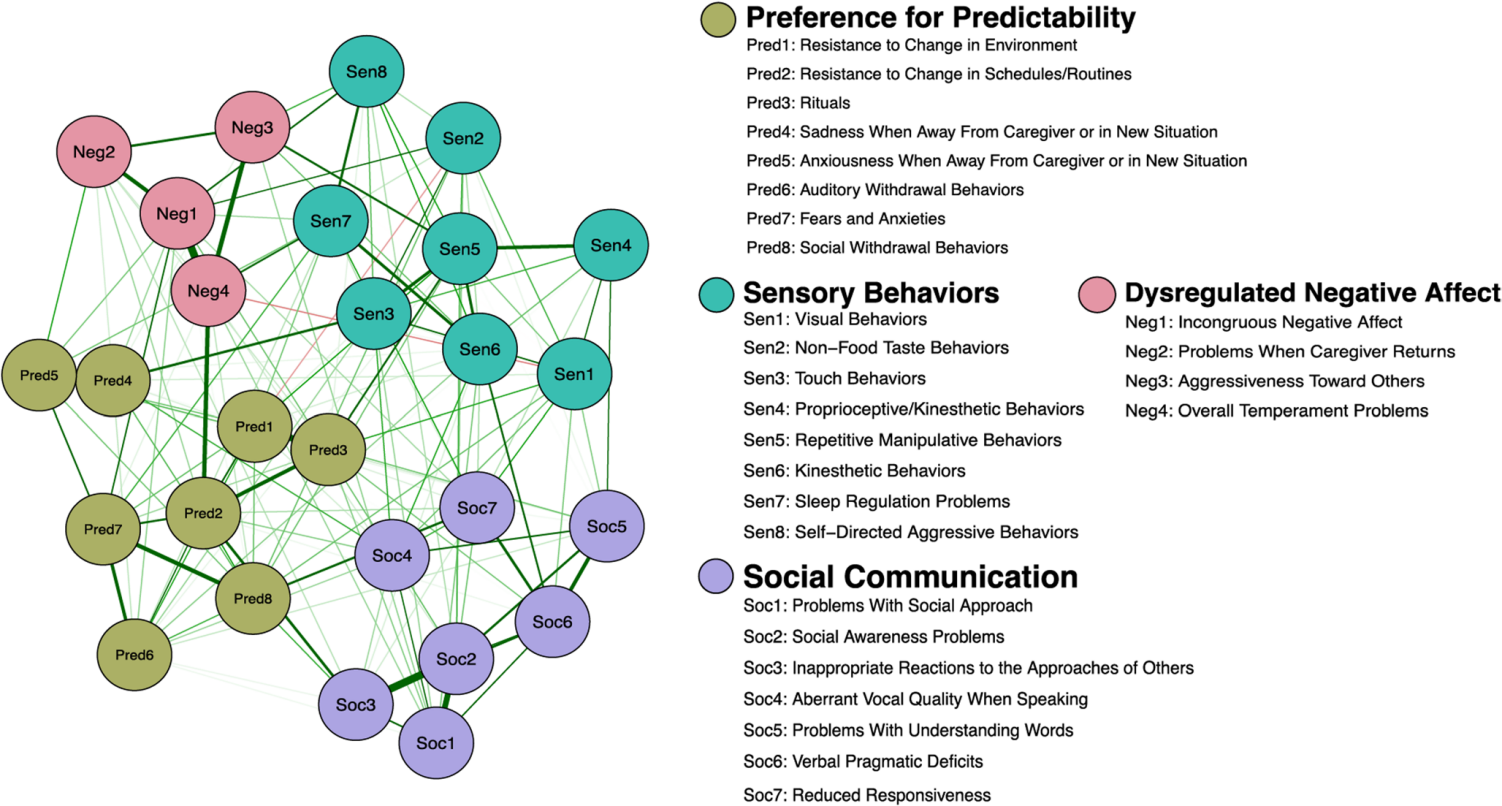
27-node approach-withdrawal network. *Note*. The network figure depicts 27 PDDBI approach-withdrawal behaviors separated into four distinct communities using the *walktrap* algorithm. Dysregulated negative affect nodes are colored pink, preference for predictability nodes are colored green, social communication nodes are colored purple, and sensory behavior nodes are colored turquoise. The network structure was determined by using the graphical LASSO coupled with EBIC model selection with $\:\gamma\:=0$0.5. Thicker edges indicate stronger relations between nodes. Green (red) edges indicate positive (negative) partial correlations

**Fig. 2:**
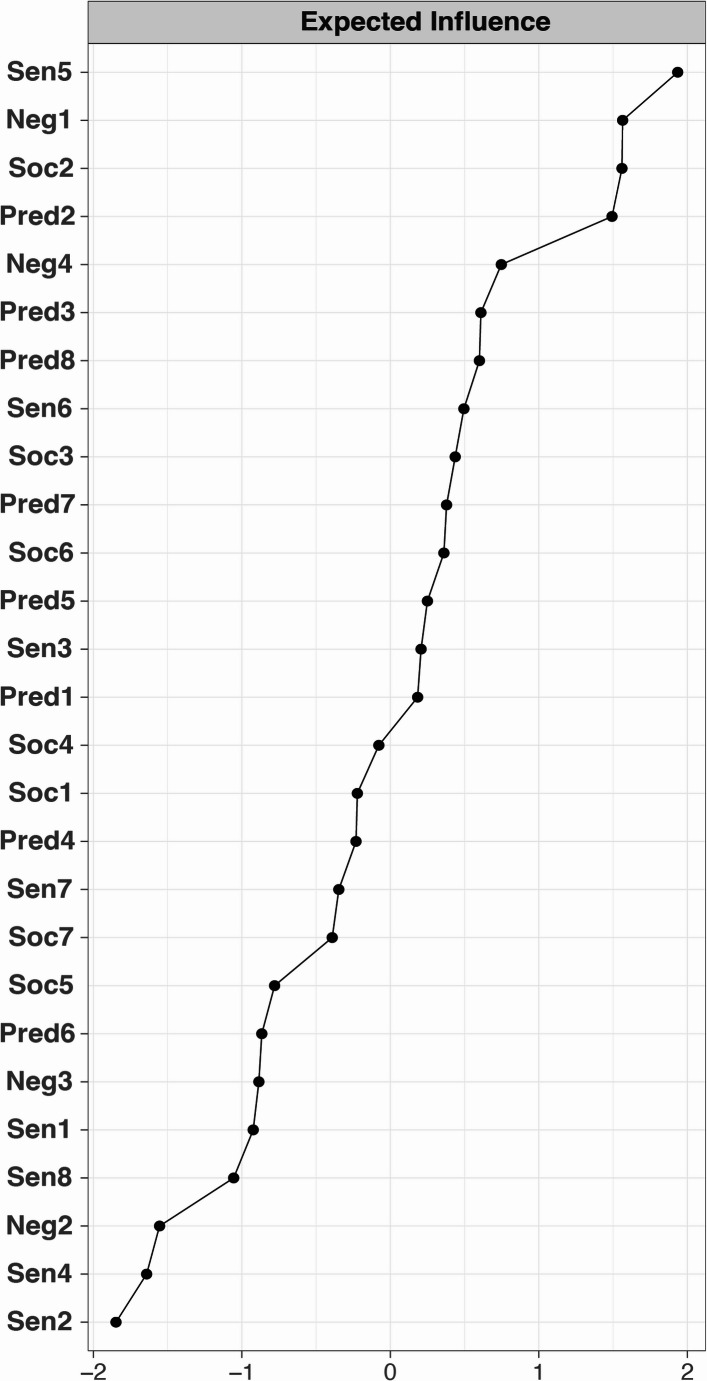
Approach-withdrawal network expected influence plot. *Note. *Standardized (Z-scored) expected influence was used to identify nodes that are most central to the network; higher values indicate greater influence (nodes with more negative expected influence are less central to the network)

The 27-node approach-withdrawal network is depicted in Fig. 1 and centrality of each node is displayed in Fig. 2. Individual nodes in the network correspond to the Approach Withdrawal Problems section of the PDDBI. The correlation stability coefficient of the expected influence was 0.37, indicating adequate stability and interpretability of centrality estimates.

### Central Nodes and Community Detection

All 27 node names are listed in descending order from most to least central in Table 2. The top third of most influential nodes (i.e., 9 of 27 nodes with the highest expected influence) are demarcated and include: repetitive manipulative behaviors (Sen5), incongruous negative affect (Neg1), social awareness problems (Soc2), resistance to change in schedules/routines (Pred2), overall temperament problems (Neg4), rituals (Pred3), social withdrawal behaviors (Pred8), kinesthetic behaviors (Sen6), and inappropriate reactions to the approaches of others (Soc3).

**Table 2:** Node legend and walktrap community (from highest to lowest node centrality)

Node Name	PDDBI Approach-Withdrawal Behavior	PDDBI Category	Walktrap Category
Sen5	Repetitive Manipulative Behaviors	Sensory/Perceptual Approach Behaviors	Sensory Behaviors
Neg1	Incongruous Negative Affect	Aggressiveness	Dysregulated Negative Affect
Soc2	Social Awareness Problems	Social Pragmatic Problems	Social Communication
Pred2	Resistance to Change in Schedules/Routines	Ritualisms/Resistance to Change	Preference for Predictability
Neg4	Overall Temperament Problems	Aggressiveness	Dysregulated Negative Affect
Pred3	Rituals	Ritualisms/Resistance to Change	Preference for Predictability
Pred8	Social Withdrawal Behaviors	Specific Fears	Preference for Predictability
**Sen6**	**Kinesthetic Behaviors**	**Arousal Regulation Problems**	**Sensory Behaviors**
Soc3	Inappropriate Reactions to the Approaches of Others	Social Pragmatic Problems	Social Communication
Pred7	Fears and Anxieties	Specific Fears	Preference for Predictability
Soc6	Verbal Pragmatic Deficits	Semantic/Pragmatic Problems	Social Communication
Pred5	Anxiousness When Away From Caregiver or in New Situation	Specific Fears	Preference for Predictability
Sen3	Touch Behaviors	Sensory/Perceptual Approach Behaviors	Sensory Behaviors
Pred1	Resistance to Change in Environment	Ritualisms/Resistance to Change	Preference for Predictability
Soc4	Aberrant Vocal Quality When Speaking	Semantic/Pragmatic Problems	Social Communication
Soc1	Problems With Social Approach	Social Pragmatic Problems	Social Communication
Pred4	Sadness When Away From Caregiver or in New Situation	Specific Fears	Preference for Predictability
**Sen7**	**Sleep Regulation Problems**	**Arousal Regulation Problems**	**Sensory Behaviors**
**Soc7**	**Reduced Responsiveness**	**Arousal Regulation Problems**	**Social Communication**
Soc5	Problems With Understanding Words	Semantic/Pragmatic Problems	Social Communication
Pred6	Auditory Withdrawal Behaviors	Specific Fears	Preference for Predictability
Neg3	Aggressiveness Toward Others	Aggressiveness	Dysregulated Negative Affect
Sen1	Visual Behaviors	Sensory/Perceptual Approach Behaviors	Sensory Behaviors
**Sen8**	**Self-Directed Aggressive Behaviors**	**Aggressiveness**	**Sensory Behaviors**
Neg2	Problems When Caregiver Returns	Aggressiveness	Dysregulated Negative Affect
Sen4	Proprioceptive/Kinesthetic Behaviors	Sensory/Perceptual Approach Behaviors	Sensory Behaviors
Sen2	Non-Food Taste Behaviors	Sensory/Perceptual Approach Behaviors	Sensory Behaviors

Note. Node names included in the network analysis (Fig. 1) are listed in the first column in descending order of expected influence, followed by the name of the specific approach-withdrawal behavior as listed in the PDDBI. Column three indicates the node’s PDDBI category. Column four indicates the node’s *walktrap* category. The rows in bold indicate nodes that clustered in a different *walktrap* community than the other nodes in its original PDDBI category

Table 2 summarizes how nodes were conceptually organized based on the PDDBI and *walktrap* algorithm and provide a way to compare categories established by the PDDBI measure and categories identified using the *walktrap* community detection algorithm. Specific nodes comprising each of the four *walktrap* communities were examined and operationalized accordingly: dysregulated negative affect, sensory behaviors, preference for predictability, and social communication. As shown in Table 2, most nodes belonging to the same PDDBI category were grouped in the same *walktrap* community. Deviations from this pattern are indicated in bold in Table 2. For example, while two of the three nodes from the arousal regulation problems PDDBI category clustered together in the sensory behaviors *walktrap* community, one (*reduced responsiveness*) was grouped into social communication based on the community detection algorithm. Additionally, four of the five nodes belonging to the aggressiveness PDDBI category clustered together in the dysregulated negative affect *walktrap* community. The fifth node, *self-directed aggressive behaviors*, was instead included in the sensory behaviors *walktrap* community.

### Associations Between Co-occurring Conditions and Central Approach-Withdrawal Nodes

**Fig. 3:**
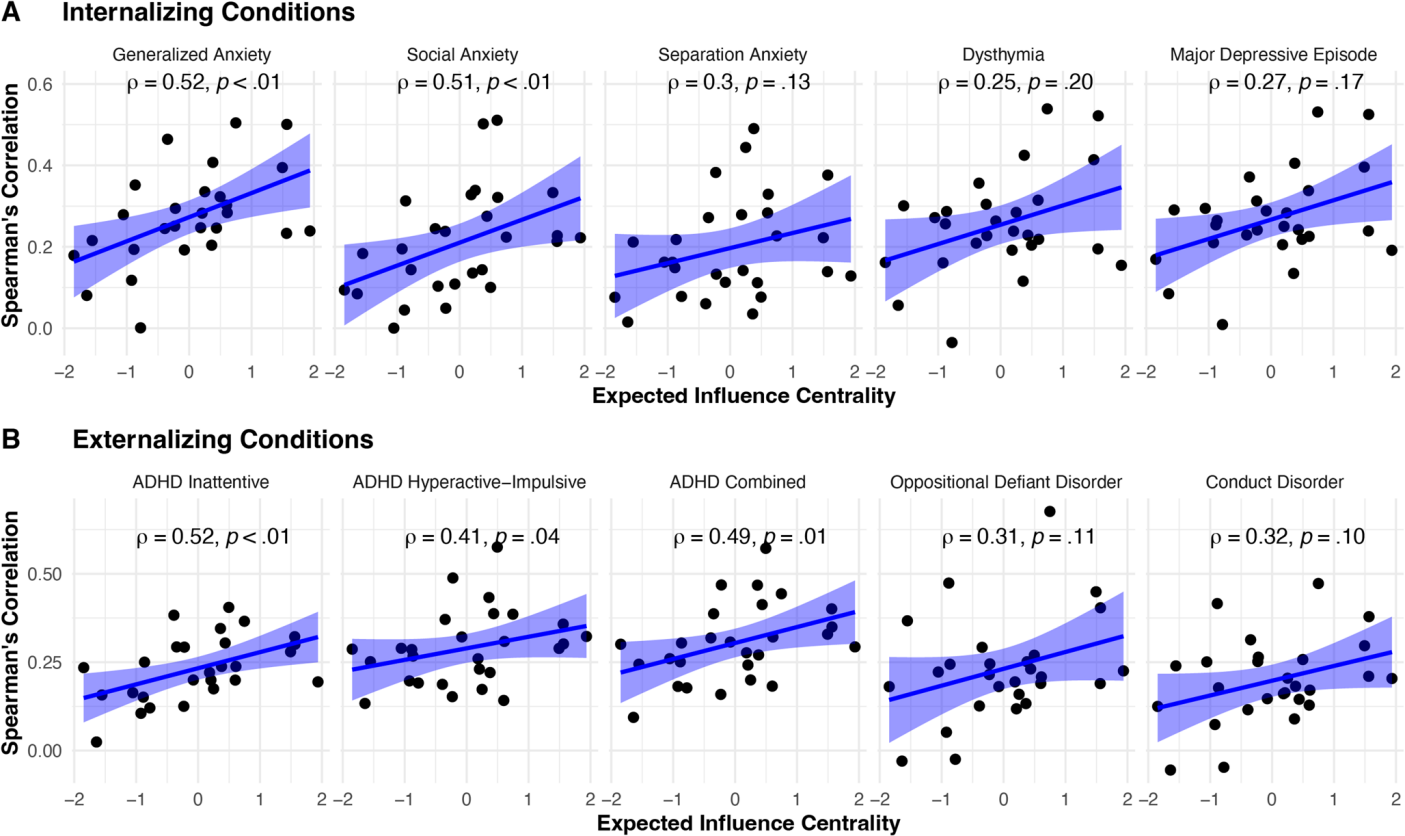
Linear regressions assessing the relationship between the approach-withdrawal network and each CASI-5 co-occurring condition. *Note. *Plots are organized by internalizing conditions (**A**) and externalizing conditions (**B**) to depict associations between node centrality and correlations between the node and the co-occurring condition. Each plot shows 27 points corresponding to the 27 approach-withdrawal behaviors. The x-axis reflects standardized expected influence node centrality and the y-axis reflects the zero-order correlations between PDDBI nodes and the CASI-5 co-occurring condition. Positive relationships indicate that autism, when considered as an interrelated network of approach-withdrawal behaviors, is associated with the co-occurring condition

Results are organized by internalizing conditions (generalized anxiety, social anxiety, separation anxiety, dysthymia, and major depressive episode) and externalizing conditions (ADHD inattentive, ADHD hyperactive-impulsive, ADHD combined type, ODD, and CD). As shown in Fig. 3, each point represents a symptom (i.e., node) in the network. A point that is toward the right on the x-axis represents a symptom that was highly central to the network. A point that is high on the y-axis represents an approach-withdrawal behavior that was highly correlated with a co-occurring condition. A positive relationship between the x-variable (node centrality) and y-variable (correlation between each node and CASI-5 condition) indicates that facets of autism-relevant approach-withdrawal behaviors, when considered in the context of a network, are associated and potentially predictive of co-occurring symptoms.

Node centrality was positively associated with all co-occurring clinical conditions assessed (though the association was not always statistically significant). Associations between central approach–withdrawal behaviors and co-occurring symptoms ranged from 0.25 to 0.52, indicating medium-to-large effects (Cohen, 2013). Relations were strongest between node centrality and scores on the CASI-5 generalized anxiety subscale ($\:\rho\:$(278) = 0.52, *p* <.01), followed by social anxiety ($\:\rho\:$(278) = 0.51, *p* <.01), ADHD inattentive type ($\:\rho\:$(278) = 0.52, *p* <.01), and ADHD combined type ($\:\rho\:$(278) = 0.49, *p* =.01).

### Differential Associations Between Approach-Withdrawal Behaviors and Co-occurring Conditions

**Fig. 4:**
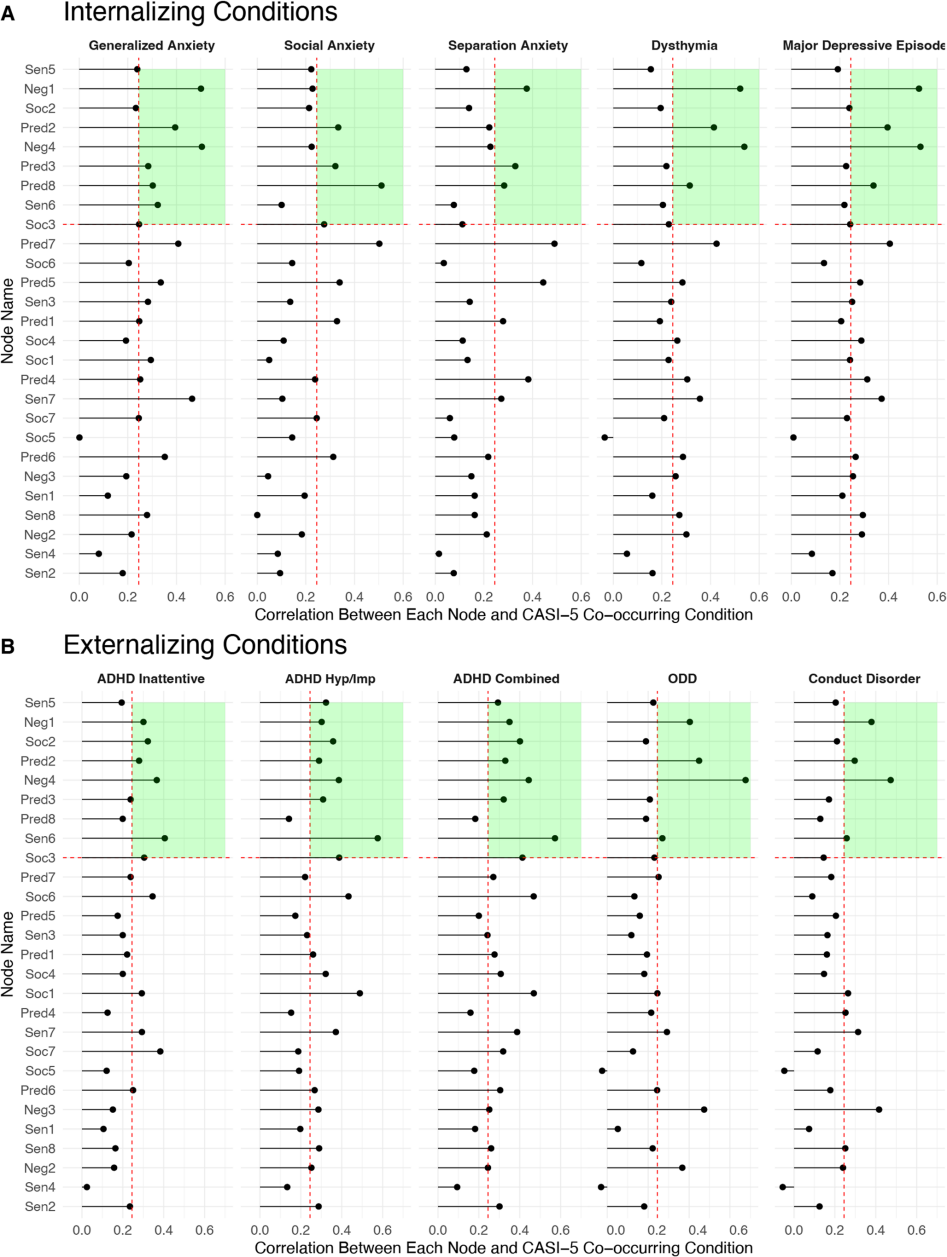
Node importance plots for (**A**) Internalizing Conditions and (**B**) Externalizing Conditions. *Note. *Plots are organized by (**A**) internalizing conditions and (**B**) externalizing conditions to depict clinically relevant nodes. The x-axis reflects zero-order correlations between PDDBI nodes and the CASI-5 co-occurring condition and the y-axis are node listed from highest (top) to lowest (bottom) expected influence. Red dashed lines demarcate the top third nodes with highest centrality and correlations exceeding the threshold of 0.25. Green shaded regions indicate above-threshold regions indicated by the dashed red lines

Node importance plots are displayed in Fig. 4 and can be interpreted in conjunction with Table 3. Figure 4 illustrates how specific facets of approach and withdrawal may be *differentially* related to internalizing conditions (Fig. 4 A) and externalizing conditions (Fig. 4B). In these plots, the x-axis reflects the zero-order correlations between PDDBI nodes and the CASI-5 co-occurring condition. The y-axis includes all 27 PDDBI facets of approach-withdrawal behaviors ranked by centrality (standardized expected influence). Nodes toward the top of the plot have the highest centrality.

**Table 3:** Clinically relevant approach-withdrawal nodes for each CASI-5 condition

CASI-5 Condition	Clinically Relevant Approach-Withdrawal Nodes
*Internalizing Conditions*	
Generalized Anxiety	• Resistance to Change in Schedules/Routines • Incongruous Negative Affect • Overall Temperament Problems
Social Anxiety	• Resistance to Change in Schedules/Routines • Social Withdrawal Behaviors
Separation Anxiety	• Incongruous Negative Affect
Dysthymia	• Resistance to Change in Schedules/Routines • Incongruous Negative Affect • Overall Temperament Problems
Major Depressive Episode	• Resistance to Change in Schedules/Routines • Social Withdrawal Behaviors • Incongruous Negative Affect • Overall Temperament Problems
*Externalizing Conditions*	
ADHD Inattentive	• Kinesthetic Behaviors • Overall Temperament Problems
ADHD Hyperactive-Impulsive	• Social Awareness Problems • Inappropriate Reactions to the Approaches of Others • Kinesthetic Behaviors • Overall Temperament Problems
ADHD Combined Type	• Social Awareness Problems • Incongruous Negative Affect • Inappropriate Reactions to the Approaches of Others • Kinesthetic Behaviors • Overall Temperament Problems
Oppositional Defiant Disorder	• Resistance to Change in Schedules/Routines • Incongruous Negative Affect • Overall Temperament Problems
Conduct Disorder	• Incongruous Negative Affect • Overall Temperament Problems

*Note.* “Clinically relevant” nodes are defined as those in the top nine most central to the 27-node approach-withdrawal network that are also correlated with the clinical condition at or above $\:\rho\:$=0.25. As a reminder, approach-withdrawal nodes were defined based on the Approach Withdrawal Problems section of the PDDBI

#### Designation of Clinically Relevant Approach and Withdrawal Behaviors

**Fig. 5:**
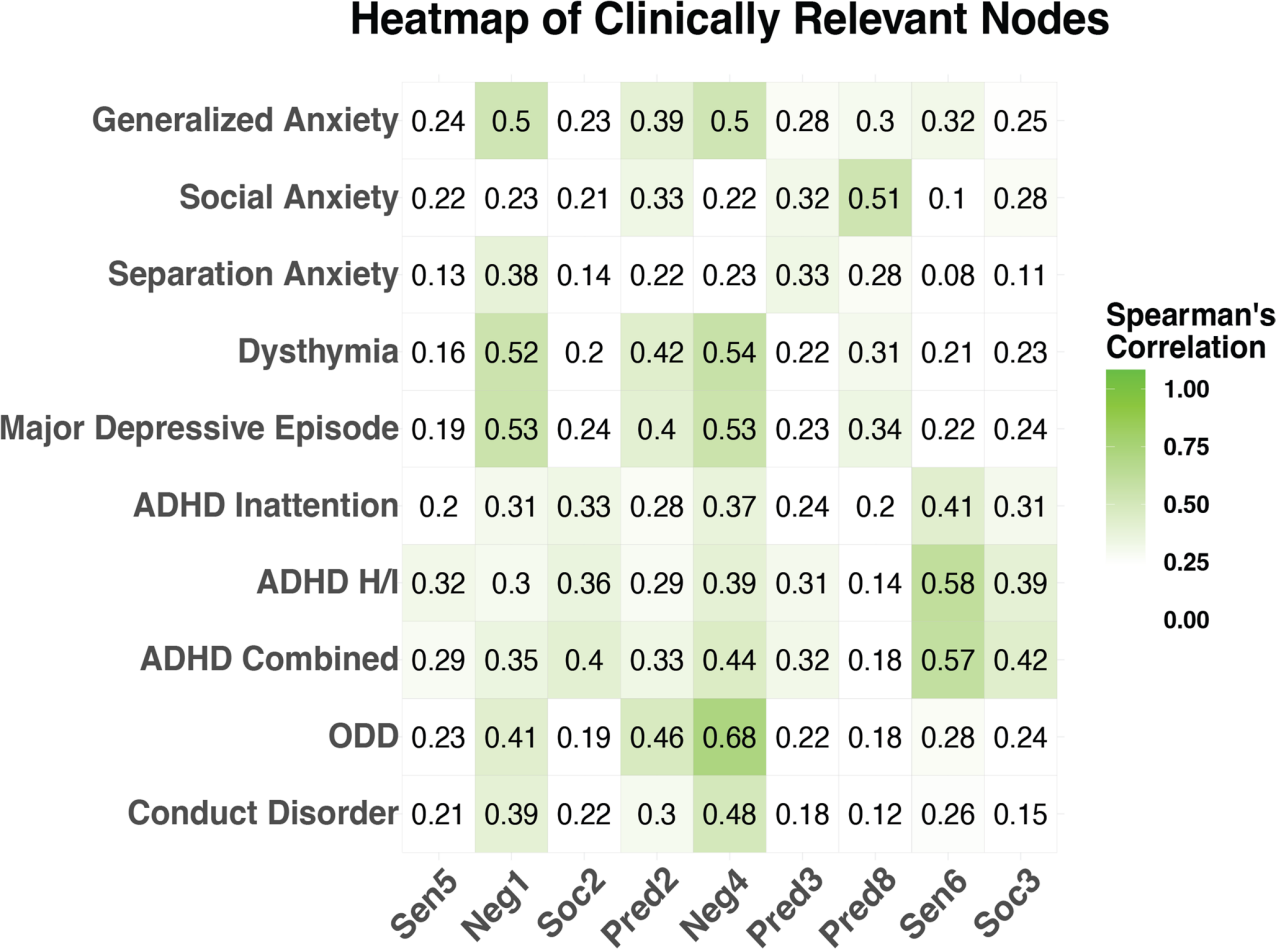
Heatmap of clinically relevant nodes for each co-occurring condition. *Note. *The heatmap provides a visual summary of nodes that are clinically relevant to each co-occurring condition; correlations are shaded green if they exceed the cutoff of 0.25. Reference Table 2 for node legend

Approach and withdrawal behaviors were operationalized as “clinically relevant” if they had high centrality in the approach-withdrawal network representing autism (Fig. 1) *and* greater association with co-occurring psychiatric symptoms. The average correlation of *all* correlations between PDDBI approach-withdrawal nodes and CASI-5 conditions was $\:\rho\:$=0.25. Thus, nodes of the network with correlations exceeding $\:\rho\:$=0.25 *and* situated in the top third of the expected influence plot (Fig. 2)—i.e., nodes that were central in the approach-withdrawal network *and* were strongly associated with the CASI-5 condition—were considered particularly relevant to the relationship between autism itself and the co-occurring condition. These are visually depicted in Fig. 4 by the green shaded region in the top right quadrant of plots corresponding to each co-occurring condition and correspond to the nodes listed in Table 3. Conversely, node associations outside of the green shaded region but exceeding $\:\rho\:$=0.25 reflect approach-withdrawal behaviors that are less central to autism but strongly associated with the co-occurring condition, thereby being particularly important for differential diagnosis (i.e., differentiating autism from the co-occurring condition). A summary of clinically relevant nodes for each condition is presented in Table 3 and visually depicted by the heatmap in Fig. 5. Incongruous negative affect (Neg1), resistance to changes in schedules/routines (Pred2), overall temperament problems (Neg4), and social withdrawal behaviors (Pred8) were consistently clinically relevant to generalized anxiety, dysthymia, and major depressive episode. Regarding externalizing conditions, incongruous negative affect (Neg1), resistance to changes in schedules/routines (Pred2), and temperament problems (Neg4) were influential autism nodes that were also consistently clinically relevant to ODD and conduct disorder. Many nodes that were highly central to the autism approach-withdrawal network were also clinically relevant to ADHD, with kinesthetic behaviors (Sen6) demonstrating a particularly strong association across ADHD subtypes.

## Discussion

Heterogeneity in autism is compounded by co-occurring psychiatric symptoms, the presence of which is associated with worse longitudinal outcomes, heightened disability and morbidity, and increased rates of suicidality. The current study investigated how transdiagnostic approach-withdrawal features of autism relate to common co-occurring conditions in a large, well-characterized pediatric sample of autistic children. To address interdependence of various autism-relevant approach-withdrawal behaviors, we utilized network analysis to understand how features of autism, when considered as a *system of symptoms*, are related to co-occurring symptomatology. While leveraging the network perspective of clinical conditions (Borsboom & Cramer, 2013), we also examined how specific approach-withdrawal features may be *differentially* related to co-occurring internalizing and externalizing symptoms. To our knowledge, this is the first study utilizing network analysis to understand relations among transdiagnostic features of autism and co-occurring symptoms across *many* clinical conditions of childhood. By applying this analytic approach, we provide a comprehensive quantitative summary of behaviors underlying psychiatric symptoms in autistic children, highlighting how DSM-5’s categorical checklist approach may obscure the motivational processes linking autism with co-occurring conditions and contribute to both over- and under-diagnosis.

### PDDBI Approach-Withdrawal Network and Community Detection

Results from a community detection algorithm revealed four “communities” of behaviors: *preference for predictability*,* sensory features*,* social communication*,* and dysregulated negative affect*. This contrasts the seven categories outlined in the PDDBI that were previously validated psychometrically via factor analysis (Cohen et al., 2003). Considering recent simulation studies indicating how community detection algorithms in the network analysis framework may complement more traditional factor-analytic approaches (Christensen et al., 2021), the re-organization of nodes via the *walktrap* algorithm offers insights into which approach-withdrawal domains may be more similar than others. Specifically, the combination of the PDDBI Ritualisms/Resistance to Change and PDDBI Specific Fears domains into one community may suggest that repetitive and inflexible behaviors are maintained by both overactive approach toward predictability and overactive withdrawal from unpredictability. The combination of PDDBI Sensory/Perceptual Approach and Arousal Regulation Problems is consistent with prior work suggesting that sensory features are maintained by a dysregulated arousal system (Cascio et al., 2016; Schaaf et al., 2015). Finally, the combination of Semantic Pragmatic and Social Pragmatic Problems suggests the inextricable link between these two facets of social functioning.

### Differential Node Importance To Clarify Symptom Overlap and Inform Differential Diagnosis

In the modifier model of autism, transdiagnostic or “modifier processes” (e.g., executive functioning, social awareness, and motivational biases) are hypothesized to alter the expression of core autism features and explain behavioral and psychological differences (i.e., co-occurring symptomatology) in autistic children (Mundy et al., 2007). By investigating autism-relevant approach-withdrawal behaviors that are also related to co-occurring conditions, our analysis identified potential modifier processes linking autism features to many types of co-occurring psychopathology. Node associations in the green shaded region are influential to autism *and* strongly associated with the co-occurring condition, indicating potential modifier processes while node associations outside of the shaded region may inform behavioral *differences* in autism compared to co-occurring conditions to facilitate differential diagnosis. Identifying which approach–withdrawal features act as modifier processes lays the groundwork for clarifying diagnostic overlap and refining intervention, which we illustrate below through node-level implications.

### Specific Diagnostic Implications from Node-Level Findings

The identification of specific approach–withdrawal nodes linked to psychiatric conditions highlights concrete risks for diagnostic overlap and overshadowing. For example, resistance to change in schedules or routines was implicated in both anxiety and disruptive behavior conditions, suggesting that the same behavior may be misinterpreted as oppositionality when it is instead rooted in anxiety-related rigidity. Similarly, social withdrawal behaviors, often attributed to core autism features, were uniquely associated with social anxiety and major depressive episodes in this network, underscoring the risk of under-diagnosis if clinicians assume withdrawal is “part of autism” rather than a marker of treatable comorbidity. For externalizing conditions, kinesthetic behaviors and arousal regulation difficulties were strongly associated with ADHD subtypes, yet these features may overlap with autism-related regulation problems, increasing the likelihood of over-diagnosis of ADHD in autistic children. Finally, the finding that overall temperament problems and incongruous negative affect cut across both internalizing and externalizing domains suggests that emotional dysregulation is a shared transdiagnostic process, and its presence alone may not distinguish autism from comorbid psychopathology. Collectively, these results emphasize that careful attention to the motivational context of specific behaviors is critical for differential diagnosis, and that a network approach may help clinicians parse whether a behavior reflects autism-related variability, a co-occurring condition, or both.

### From Diagnostic Clarity to Intervention Targets

Beyond advancing diagnostic clarity, these findings have direct implications for the contexts in which autistic children live and learn. Families and educators are often the first to notice difficulties such as withdrawal, rigidity, impulsivity, or disruptive behaviors, yet may interpret them differently depending on the setting. By situating these behaviors within an approach–withdrawal framework, clinicians, teachers, and caregivers can better distinguish autism-related variability from co-occurring symptoms and coordinate supports across home, school, and clinic settings.

### Approach–Withdrawal Features in Internalizing Conditions: Implications for Diagnosis and Intervention

With respect to internalizing conditions, incongruous negative affect (Neg1), overall temperament problems (Neg4), and resistance to changes in schedules/routines (Pred2) were consistently clinically relevant to generalized anxiety, dysthymia, and depression, while social withdrawal behaviors (Pred8) was most relevant to social anxiety. Results suggest an association between resistance to changes in schedules and problems with affect regulation and temperament. This is consistent with literature investigating the role of *intolerance of uncertainty* (Dugas et al., 2004), a cognitive process characterized by increased discomfort in response to unpredictability and ambiguity, and its mediating role between autism and co-occurring anxiety (Boulter et al., 2014). Persistent deviations between expectations and outcome (i.e., uncertainty) have been described as an aversive experience for autistic individuals and may elicit heightened stress and anxiety throughout the day (Simon & Corbett, 2013; Tomarken et al., 2015), especially in response to unexpected events. This stress cascade can then maintain greater negative affect and overall temperament problems, leading to elevated internalizing symptoms (Yarger & Redcay, 2020).

Building on this, interventions that increase flexibility and tolerance of uncertainty are likely to be particularly beneficial. An approach–withdrawal framework further clarifies when different intervention strategies may be most effective: difficulties rooted in overactive approach toward predictability (e.g., rigidity, resistance to change) may respond best to behavioral strategies such as flexibility training or graded exposure to uncertainty. In contrast, withdrawal-related difficulties characterized by heightened negative affect may require psychosocial interventions that emphasize emotion regulation and coping, such as cognitive-behavioral strategies targeting emotional awareness, reappraisal, and stress management. Thus, identifying whether a child’s difficulties stem from rigid approach tendencies or from heightened withdrawal and negative affect can help clinicians determine whether behavioral modification or emotion-regulation–focused interventions are the most appropriate pathway. These insights may also extend to medical comorbidities in autism, which we discuss further in Future Directions.

These distinctions also extend beyond psychiatric comorbidity to the high rates of co-occurring medical conditions in autism. Stress and uncertainty often exacerbate challenges such as sleep disruption, gastrointestinal difficulties, and chronic pain. In these contexts, behavioral modification strategies (e.g., structured sleep routines, reinforcement-based adherence supports, graded exposure for pain-related avoidance) may be most effective when difficulties reflect rigid approach tendencies. Conversely, psychosocial interventions emphasizing affect regulation and coping may be particularly relevant when avoidance and negative affect contribute to medical difficulties. In this way, conceptualizing autism and co-occurring conditions through an approach–withdrawal framework not only clarifies psychiatric comorbidity but also highlights intervention targets that are transdiagnostically relevant across psychiatric and medical domains, supporting more holistic approaches to autistic wellbeing.

### Approach-Withdrawal Features in Externalizing Conditions: Implications for Diagnosis and Intervention

Consistent with prior work suggesting that arousal and self-regulation problems are implicated in both autism and ADHD, kinesthetic behaviors (Sen6) and overall temperament problems (Neg4) were consistently identified as clinically relevant to inattentive, hyperactive-impulsive, and combined subtypes. Social awareness problems (Soc2) and inappropriate reactions to peers—difficulties well-documented in both ADHD and autism (Han et al., 2020; Humphreys et al., 2016)—were especially relevant to the combined subtype. These findings highlight a diagnostic challenge: kinesthetic behaviors and arousal regulation problems may mimic ADHD symptomatology, leading to potential over-diagnosis when these difficulties actually stem from autism-related regulation issues. Conversely, impairments in social awareness and responses to peers may reflect a co-occurring ADHD phenotype, suggesting that careful attention to motivational context is essential for accurate diagnosis.

From a treatment perspective, this distinction has important implications. When externalizing symptoms reflect autism-related regulatory challenges, behavioral interventions targeting arousal regulation (e.g., structured activity breaks, sensory modulation strategies) may be sufficient. By contrast, when symptoms stem from ADHD-related impulsivity and reduced inhibition, interventions may need to emphasize skills for self-monitoring, delay of gratification, and cognitive control. Social awareness difficulties that contribute to disruptive behaviors highlight the importance of social skills interventions and approaches focused on building emotional insight and regulation that can be implemented across home and school settings.

Finally, incongruous negative affect (Neg1) and overall temperament problems (Neg4) emerged as central nodes that were also implicated in ODD and CD symptoms. Of note, aggressiveness toward others (Neg3) exceeded the correlation threshold for both conditions but was not highly central to the approach-withdrawal network. This may suggest a feature of approach-withdrawal behaviors that is more distinctly related to ODD and CD and is less “core” to autism. Notably, resistance to change in routines (Pred2) was clinically relevant in ODD but to a lesser extent in CD.

Disruptive behaviors in ODD are often preceded by demands from the caregiver or environment. Demands requiring flexibility and deviations from expectation may be particularly difficult to experience for autistic individuals, leading to behavioral dysregulation. Therefore, treatments focused on increasing skills for tolerating uncertainty, improving behavioral flexibility, and effectively communicating wants and needs may be particularly helpful to reduce ODD symptoms in autistic children. These findings suggest that interventions for ODD symptoms in autistic children should not only focus on behavioral compliance but also on improving tolerance for uncertainty, building flexibility, and equipping children with effective communication strategies to express frustration. In cases where negative affect is central, affect-regulation interventions (e.g., emotion identification, coping strategies, parent-mediated emotion coaching) may be particularly beneficial.

### Limitations and Future Directions

Despite the well-characterized sample and innovative analytic approach, this study is not without limitations. First, causal conclusions are limited by the cross-sectional nature of the study and results can only demonstrate how a network of autism-related approach-withdrawal behaviors relates to concurrent psychiatric symptomatology for autistic children between the ages of 6 and 11 years. Longitudinal extension of this work into adolescence would allow for the identification of *prognostic* nodes at baseline that predict symptomatology at future developmental stages when clinical conditions become more relevant and prevalent. Examining temporal stability of the network structure and understanding how clinically relevant nodes change over time would also inform precision in the timing and delivery of effective treatments to target influential nodes or modifier processes linked to co-occurring psychiatric conditions. Additionally, the responses on the PDDBI and CASI-5 were completed by parent/caregiver report and were not corroborated by a trained clinician, a teacher, or self-report. Though the sample included some racial and ethnic diversity, White participants were overrepresented, underscoring the need for replication in more racially and culturally diverse samples and for culturally responsive assessment of co-occurring psychopathology. The results of the current investigation are also primarily applicable to autism defined behaviorally and do not explicitly examine the relations between syndromic manifestations of autism and co-occurring symptoms. Thus, including clinical observations from multiple informants, inclusion of more objective neurophysiological measures of approach-withdrawal behavior (e.g., alpha asymmetry (Coan & Allen, 2003; Schiltz et al., 2018) and/or genetic markers of autism, especially longitudinally, would offer a more comprehensive biobehavioral perspective on the emergence of co-occurring psychiatric conditions in autism.

An important future direction for this framework is to extend it beyond psychiatric conditions to the high rates of co-occurring medical conditions in autism, including sleep difficulties, gastrointestinal disorders, and chronic pain. These conditions contribute significantly to healthcare utilization and overall wellbeing in autistic children (e.g., Dizitzer et al. (2020). Given their prevalence and impact, applying an approach–withdrawal framework may help identify behavioral and motivational processes (e.g., avoidance of pain-related cues, over-approach to routines that interfere with sleep) that link autism features to medical challenges. Such integration would be especially relevant in pediatric psychology, where interventions that target both psychiatric and medical comorbidities could improve holistic outcomes. For example, behavioral modification strategies such as structured sleep routines, reinforcement-based adherence supports, or graded exposure for pain-related avoidance may be particularly effective when difficulties reflect rigid approach tendencies. Conversely, psychosocial interventions emphasizing affect regulation and coping may be more relevant when avoidance and negative affect contribute to medical difficulties. Together, this line of work highlights the potential for an approach–withdrawal framework not only to clarify psychiatric comorbidity but also to identify intervention targets that are transdiagnostically relevant across psychiatric and medical domains. In doing so, it offers a pathway toward more integrated, holistic approaches to autistic wellbeing.

## Conclusions

This network analysis study provides a comprehensive quantitative summary of how approach-withdrawal facets of autism may differentially relate to various clinical conditions. To our knowledge, this study is the first to consider the relationship between autism and many co-occurring conditions with this approach. By examining how individual network nodes differentially relate to internalizing and externalizing conditions, results clarify phenotypic variability in autism and point to more precise treatment strategies to reduce the negative impact of co-occurring symptomatology.

## Data Availability

ABC-CT data are publicly available via the National Database for Autism Research https://ndar.nih.gov/, #2288.
